# Dual-labeled nanoparticles based on small extracellular vesicles for tumor detection

**DOI:** 10.1186/s13062-022-00345-7

**Published:** 2022-11-14

**Authors:** Ana Santos-Coquillat, Desiré Herreros-Pérez, Rafael Samaniego, María Isabel González, Lorena Cussó, Manuel Desco, Beatriz Salinas

**Affiliations:** 1 Unidad de Medicina y Cirugía Experimental, Instituto de Investigación Sanitaria Gregorio Marañón (IiSGM), 28007 Madrid, Spain; 2 Unidad de Imagen Avanzada, Centro Nacional de Investigaciones Cardiovasculares (CNIC), 28029 Madrid, Spain; 3 Unidad de Microscopía Confocal, Instituto de Investigación Sanitaria Gregorio Marañón (IiSGM), 28007 Madrid, Spain; 4 Centro de Investigación Biomédica en Red de Salud Mental (CIBERSAM), 28029 Madrid, Spain; 5 Departamento de Bioingeniería E Ingeniería Aeroespacial, Universidad Carlos III de Madrid, 28911 Madrid, Spain

**Keywords:** Extracellular vesicles, SPECT, Oncology, Optical imaging, Diagnosis, Molecular imaging

## Abstract

**Background:**

Small extracellular vesicles (sEVs) are emerging natural nanoplatforms in cancer diagnosis and therapy, through the incorporation of signal components or drugs in their structure. However, for their translation into the clinical field, there is still a lack of tools that enable a deeper understanding of their in vivo pharmacokinetics or their interactions with the cells of the tumor microenvironment. In this study, we have designed a dual-sEV probe based on radioactive and fluorescent labeling of goat milk sEVs.

**Results:**

The imaging nanoprobe was tested in vitro and in vivo in a model of glioblastoma. In vitro assessment of the uptake of the dual probe in different cell populations (RAW 264.7, U87, and HeLa) by optical and nuclear techniques (gamma counter, confocal imaging, and flow cytometry) revealed the highest uptake in inflammatory cells (RAW 264.7), followed by glioblastoma U87 cells. In vivo evaluation of the pharmacokinetic properties of nanoparticles confirmed a blood circulation time of ~ 8 h and primarily hepatobiliary elimination. The diagnostic capability of the dual nanoprobe was confirmed in vivo in a glioblastoma xenograft model, which showed intense in vivo uptake of the SEV-based probe in tumor tissue. Histological assessment by confocal imaging enabled quantification of tumor populations and confirmed uptake in tumor cells and tumor-associated macrophages, followed by cancer-associated fibroblasts and endothelial cells.

**Conclusions:**

We have developed a chemical approach for dual radioactive and fluorescent labeling of sEVs. This methodology enables in vivo and in vitro study of these vesicles after exogenous administration. The dual nanoprobe would be a promising technology for cancer diagnosis and a powerful tool for studying the biological behavior of these nanosystems for use in drug delivery.

**Graphical Abstract:**

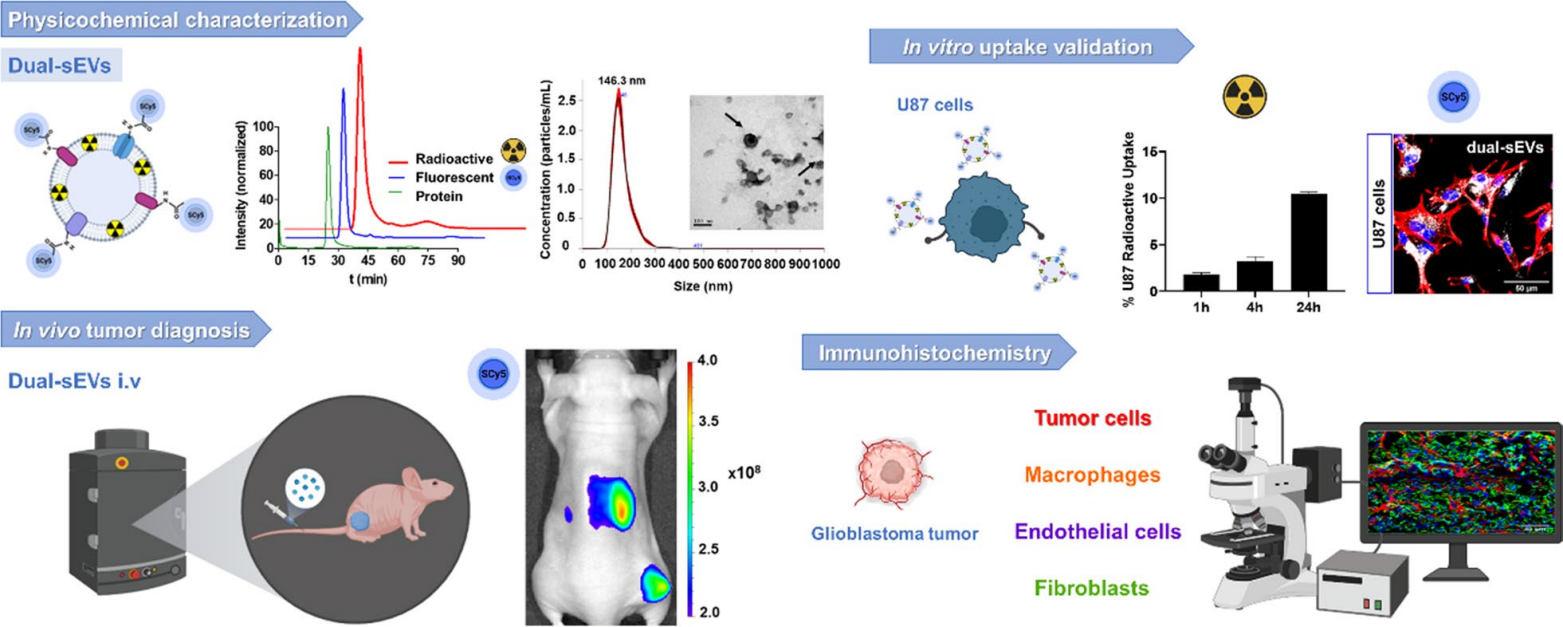

**Supplementary Information:**

The online version contains supplementary material available at 10.1186/s13062-022-00345-7.

## Background

Biomedical imaging has emerged as a revolutionary tool for detecting several pathologies, especially in oncology [1, 2]. A new line of research focused on the development of multimodal probes has gained strength and impact in the last few years [3–5]. Multimodal probes can combine several techniques in a single imaging agent, overcoming the possible limitations of each individual technique, such as the limited penetration of optical imaging and the macroscopic resolution of nuclear imaging [6]. Multimodal strategies enable the combination of in vivo imaging with ex vivo techniques, such as histopathology or autoradiography [7], providing more comprehensive, multiscale information. The detection of changes at the cellular level by optical imaging can be linked to the changes observed at the tissue level by nuclear imaging, where target specificity is critical to the efficacy of probes designed for cancer diagnosis [8]. Regarding sensitivity, the use of radioactivity and fluorescence could be considered the most valuable combination in multimodal molecular imaging [9].

Extracellular vesicles (EVs), especially small extracellular vesicles (sEVs) or exosomes, are ideal candidates for the development of novel diagnostic imaging agents due to their active role in oncological processes, small size, and biocompatibility. sEVs are nanometric EVs (30–150 nm) with the capacity for cell-to-cell communication through the delivery of biological cargo (e.g., proteins, lipids, and nucleic acids) [10]. In contrast to non-natural nanometric vehicles, such as liposomes, sEVs present distinct advantages in their use as drug delivery systems (DDS), including their natural roles in diverse biological processes [11] and their ability to naturally deliver the components of their membrane and cytoplasm by merging with the target cell membrane [12].

Due to their active role in tumor and metastasis progression [13, 14], one of the main applications of these natural nanoplatforms is oncology, either as diagnostic tools [15, 16] or as therapeutic systems intended to improve the release of chemotherapeutics in targeted tissues or processes [12, 17]. Although the preclinical applications of sEVs have been widely studied [18, 19], clinical translation still requires a deeper understanding of their largely unknown in vivo properties and behavior. This lack of information highlights the need for new tools that provide more detailed knowledge of in vivo behavior and biological interactions at the tissue and cellular levels.

Different approaches have been proposed for the development of novel EV-based probes. Among these approaches, those based on radioactive isotope labeling, such as positron emission tomography (PET) [20, 21] or single photon emission computed tomography (SPECT), stand out [22–25]. In addition, other strategies have been developed to label EVs with non-ionizing sources, such as dyes and fluorophores, including lipophilic [26], luminal [27], and engineered optical reporters [28], or by covalent bond [29]. Other strategies include magnetic resonance imaging, bioluminescence, photoacoustic imaging, or Raman labeling [30]. However, to the best of our knowledge, no dual-labeling strategy comprising nuclear and fluorescence labeling has been implemented to date.

Here, we present the synthesis of a dual (nuclear and optical) nanoprobe (dual-sEVs) based on natural milk sEVs and its evaluation as a diagnostic tool in an animal model of glioblastoma.

## Results

**Fig. 1:**
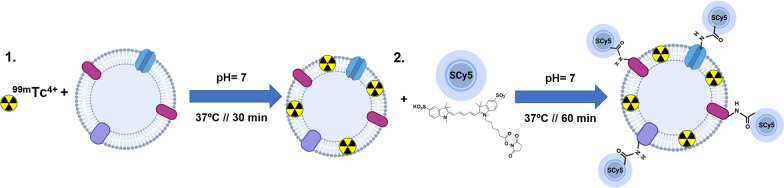
Synthesis of dual-sEVs. **1** Radioactive labeling of sEVs with ^99m^Tc (IV). **2** Fluorescent labeling of the resulting product with SCy5 fluorophore. Illustration made with Biorender

Isolated sEVs were radiolabeled with reduced ^99m^Tc (IV), and then fluorescently labeled with sulfo-cyanine5 NHS ester (SCy5; Fig. 1). A prior synthesis optimization was performed helping to settle the optimal pH, temperature, reaction time and purification steps. Based on the final activity of the probe (0.8 mCi), in 90 ug employed for radiolabelling, the resulting dual-sEVs had a specific activity of 8.8 mCi/mg and a final reaction yield of 39% ± 4.6% (n = 8).

### Physicochemical characterization

**Fig. 2:**
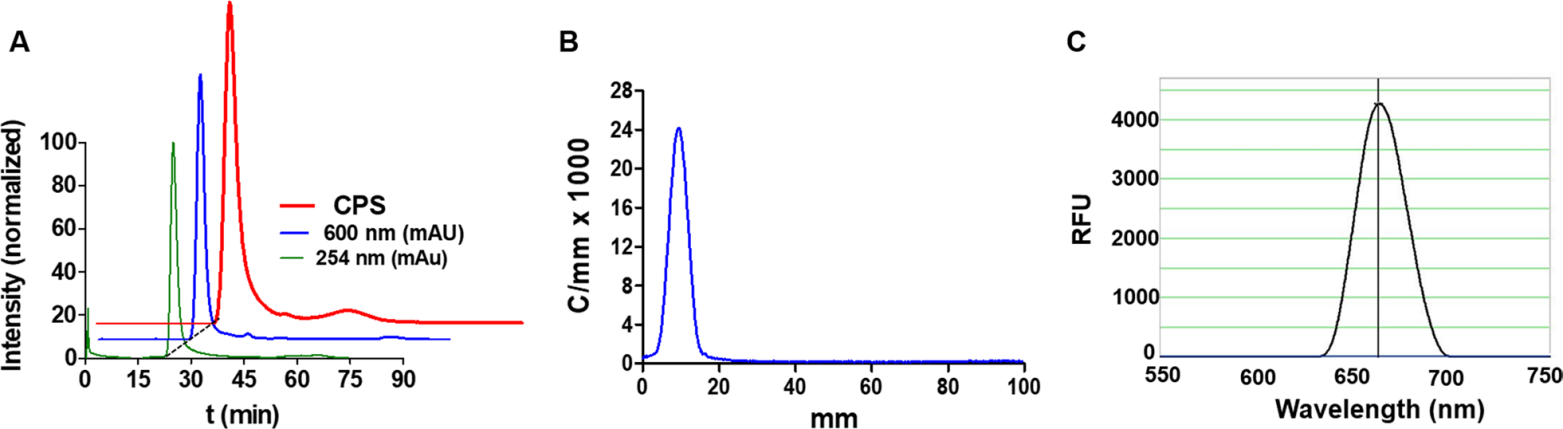
Physicochemical characterization of dual-sEVs. **A** Radioactive (red) and UV/VIS (blue) HPLC chromatograms of dual-sEVs (normalized intensity), and UV/VIS HPLC chromatogram of non-labeled sEVs (green). **B** Radio TLC chromatogram (counts/mm × 1000) of dual-sEVs. **C** Fluorimetric analysis (relative fluorescent units; RFU) of the maximum emission peak of dual-sEVs

High performance liquid chromatography (HPLC) confirmed the high purity of dual-sEVs (> 95%) at 600 nm (optical) and counts per second (CPS) (radioactive), showing a single peak at the same retention time as unlabeled sEVs (25 min) and without the peaks corresponding to free dye at ~ 75 min and free ^99m^TcO_2_ at ~ 90 min (Fig. 2A). Radio thin layer chromatography (TLC) also showed a pure product, with 100% radiochemical purity and no signal for oxidized Tc (VII) (Fig. 2B). Nanodrop fluorescence analysis showed almost 4500 RFU and a maximum emission peak at 663 nm, matching the SCy5 fluorophore specifications (Fig. 2C).

**Fig. 3:**
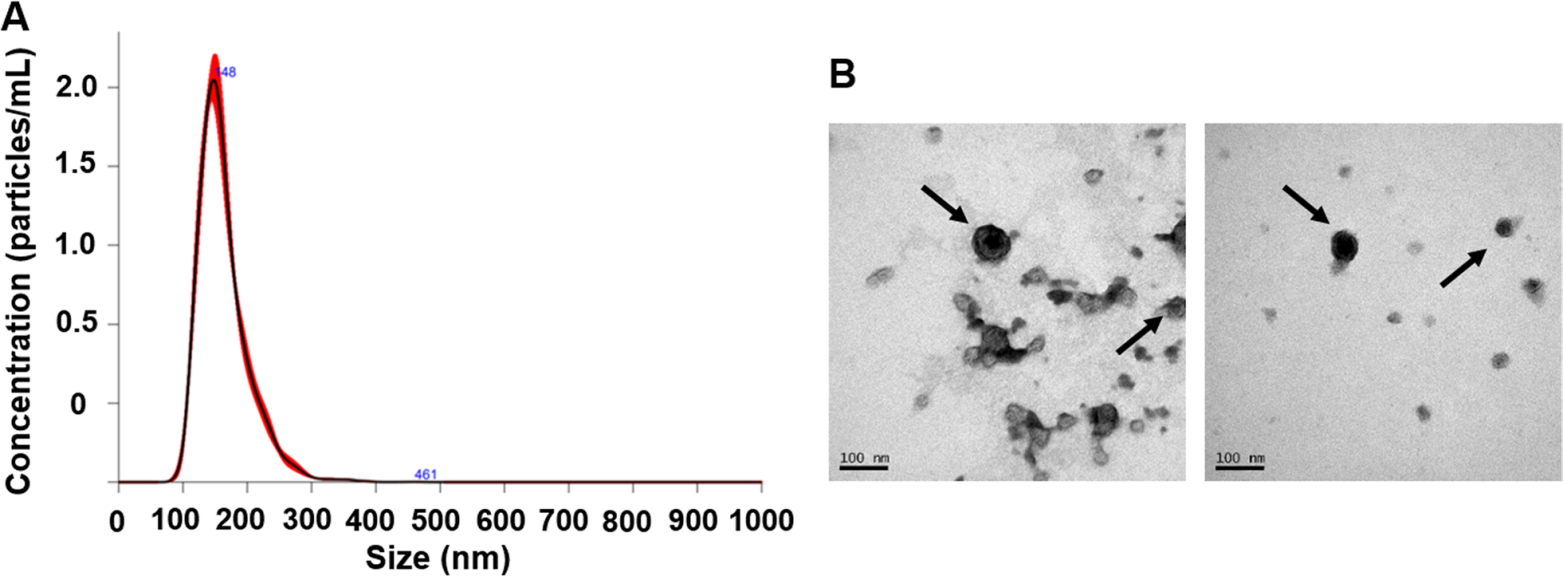
Physicochemical characterization of dual-sEVs. **A** Nanoparticle tracking analysis with concentration (particles/mL) and size of labeled sEVs (nm). **B** Transmission electron microscopy images showing the morphology and size of the sEVs

The main morphological properties of sEVs were assessed by physicochemical techniques. Nanoparticle tracking analysis (NTA) showed a homogenous population with a hydrodynamic size of 146.3 ± 3 nm (mode) and concentration of 1.42 × 10^9^ ± 4.74 × 10^8^ particles per mL after dual labeling (Fig. 3A). Control non-labeled sEVs presented a medium size of 124.44 ± 8.54 nm (Additional file 1: Fig. S1A). Therefore, the dual functionalization of these sEVs led to a size increase of approximately 20 nm. TEM confirmed the cup-shaped structure of dual-sEVs (Fig. 3B) agreed with non-labeled sEVs shape (Additional file 1: Fig. S1).

### In vitro evaluation

**Fig. 4:**
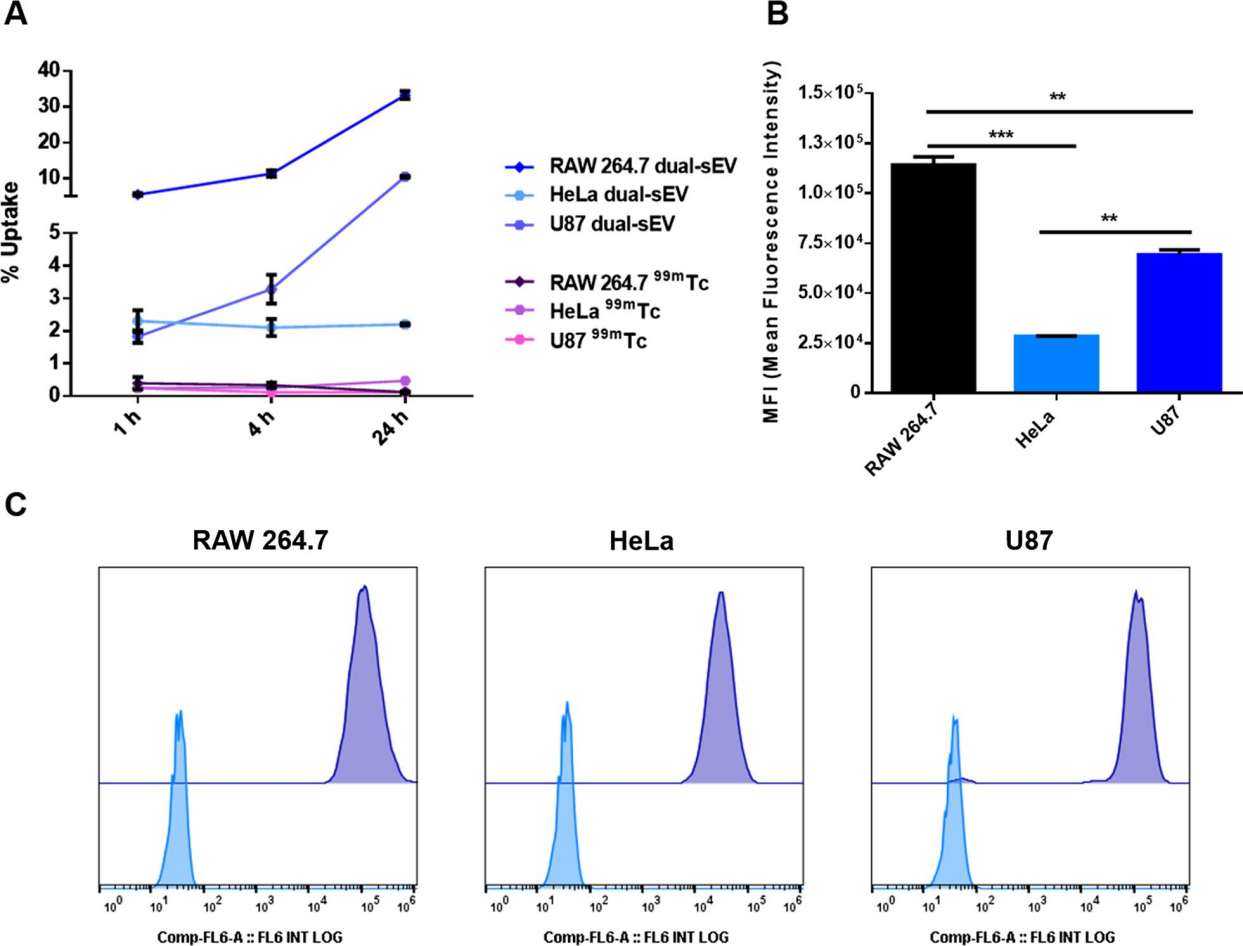
In vitro assessment of radioactive and optical uptake of dual-sEVs by gamma counter and flow cytometry. **A** Radioactive uptake for RAW 264.7, HeLa, and U87 cells after 1 h, 4 h, and 24 h of dose addition. ^99m^Tc was used as a control. Data are represented as mean ± standard deviation (SD). **B** Median fluorescence intensity (MFI) inside the cells was evaluated at 24 h. * *p* < 0.05, ** *p* < 0.01, *** *p* < 0.001. Data are represented as mean ± SD. **C** Flow cytometry diagrams of control cells (RAW 264.7, HeLa, and U87 cells, in blue) and treated cells (RAW 264.7, HeLa, and U87 cells, in purple)

We evaluated the in vitro qualitative and quantitative uptake by the different cell lines based on both nuclear and optical measurements. To assess the radioactive uptake, we employed commercial pertechnectate (^99m^Tc) as a control condition, which resulted in values < 0.5% of the maximum uptake at all evaluated time points (1 h, 4 h, and 24 h) and for all cell types (Fig. 4A). RAW 264.7 and U87 cells showed increased uptake over time, reaching the highest uptake values of ~34% in RAW 264.7 cells and ~10.5% in U87 cells after 24 h of incubation, whereas HeLa cells had an uptake of ~2% of the total radioactive dose at all time points (1 h, 4 h, and 24 h).

Optical uptake of the dual probe in the different cell lines was assessed by flow cytometry and confocal microscopy. Flow cytometry was performed at the endpoint (24 h). For the median fluorescence intensity (MFI), RAW 264.7 cells presented significantly higher values (One-way ANOVA and Tukey’s post-test; ** *p* < 0.01 for U87 cells and *** *p* < 0.001 for HeLa cells; Fig. 4B). Control cells were evaluated to check the autofluorescence of each cell type in the APC channel. Histograms in Fig. 4C show the control cells in pale blue and treated cells in purple; RAW 264.7 and HeLa cells were 100% positive for the SCy5 signal, with a clear single peak. For U87 cells, two positive populations were found: a high uptake population and a small population with a reduced fluorescence signal.

**Fig. 5:**
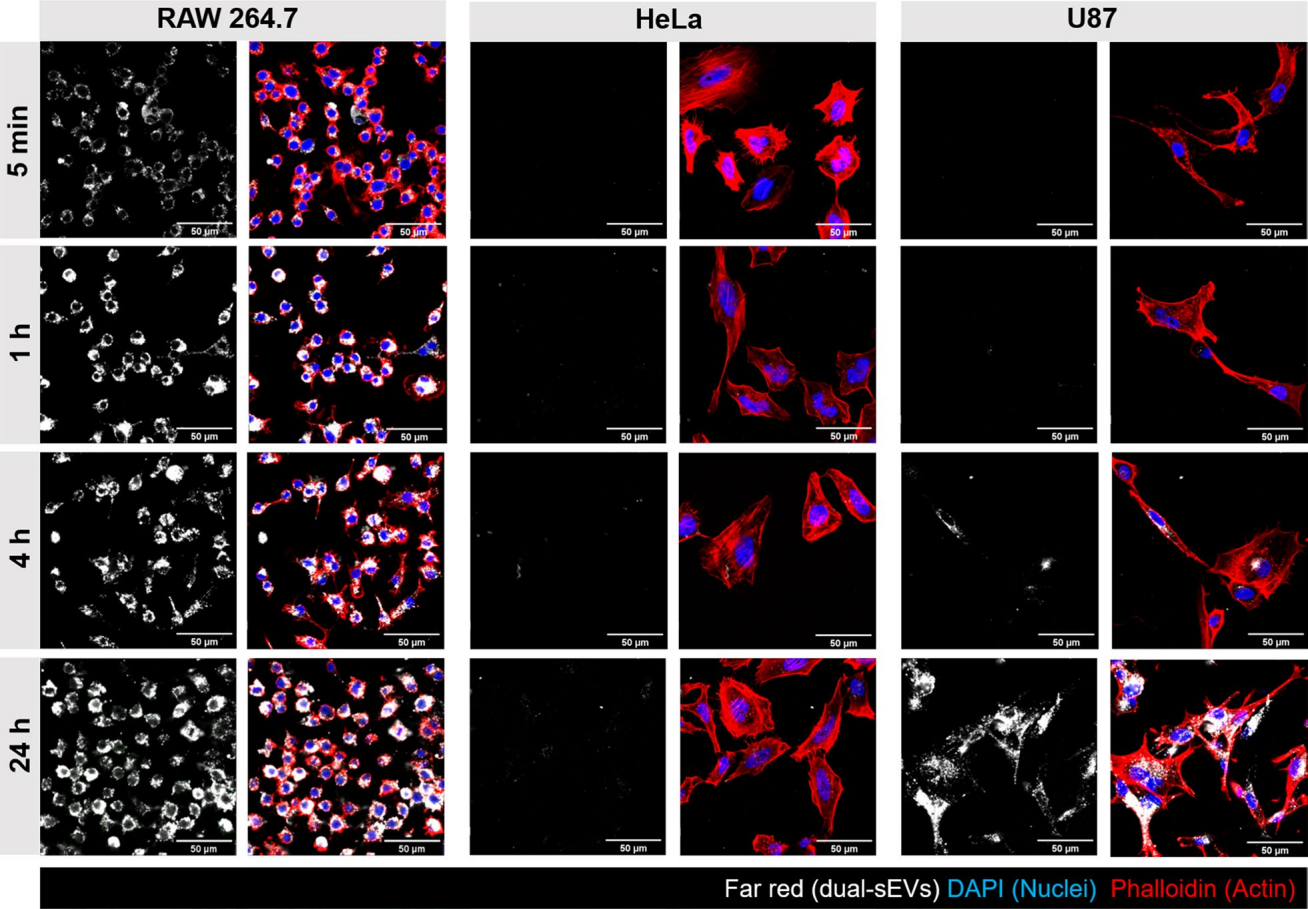
Optical uptake of dual-sEVs by confocal microscopy in RAW 264.7, HeLa, and U87 cells at 5 min, 1 h, 4 h, and 24 h after the administration of 5 μg/mL of dual-sEVs. Blue, DAPI; red, phalloidin; and white, dual-sEVs

This uptake pattern was confirmed by confocal imaging at 5 min, 1 h, 4 h, and 24 h of probe incubation (Fig. 5). HeLa cells presented a similar temporal pattern of uptake (1, 4, and 24 h) correlating with the radioactive uptake, in which they internalized 2% of the total dose. On the other hand, glioblastoma cells presented with increasing signal from 4 to 24 h.

Therefore, confocal imaging confirmed the highest uptake values for dual-sEVs in RAW 264.7 cells, followed by U87 glioblastoma cells and HeLa cells for both nuclear and optical evaluation.

### Nuclear and fluorescent in vivo assessment of dual-sEVs

**Fig. 6:**
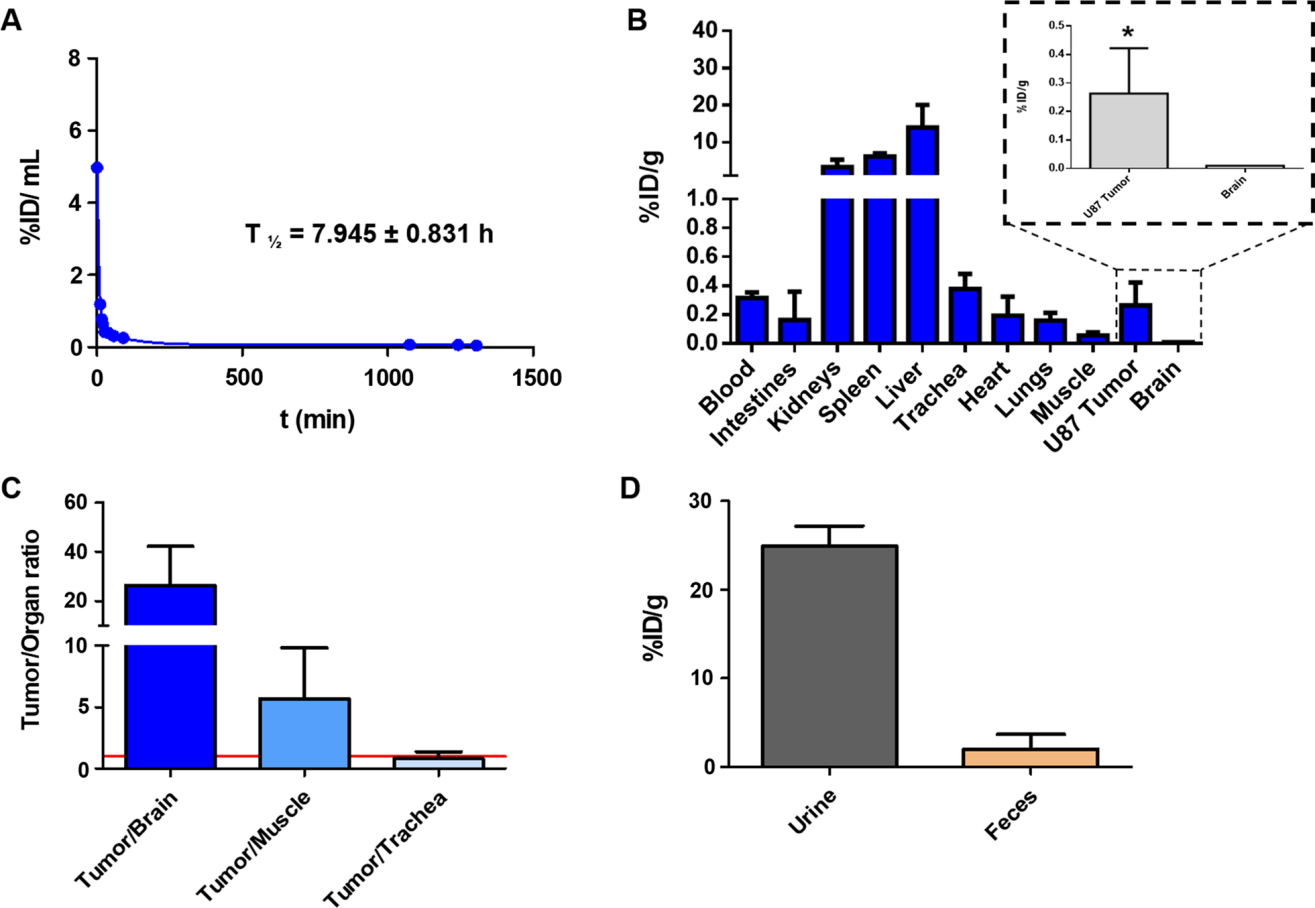
In vivo and ex vivo assessment of dual-sEVs by nuclear techniques. **A** Blood half-life. **B** Ex vivo biodistribution of dual-sEVs in a U87 xenograft mouse model 24 h after tracer injection. Detailed radioactivity in the brain (control organ) compared to U87 tumor tissue. * *p* < 0.05. **C** Ratio of dual-SEV uptake in U87 tumor tissue to non-target tissues at 24 h. **D** Excretion profile of dual-sEVs in urine and feces collected from the animal 24 h post-injection. Radioactivity in tissues is expressed as % ID/g. Data are represented as mean ± SD

Based on the results obtained in the in vitro assessment of dual-sEVs, further in vivo validation of the probe as a diagnostic tool in oncology was performed in a xenograft mouse model of glioblastoma. In vivo blood half-life (t_1/2_ β) analysis found a t_1/2_ β of 7.9 ± 0.8 h and a blood clearance (CL) of 21.0 ± 4.7 mL/day/g (Fig. 6A). Quantitative ex vivo biodistribution studies based on the radioactive signal revealed a high uptake of the probe in reticuloendothelial and excretory organs, such as the liver (13.94 ± 6.11% ID/g), spleen (6.12 ± 0.82% ID/g), and kidneys (3.25 ± 2.04% ID/g). Glioblastoma U87 tumor cells (TCs) had a median uptake 26-fold higher than healthy control brain tissue (paired t-test; * *p* < 0.05, Fig. 6B). Moreover, tumor/organ activity ratios for brain, muscle, and trachea (Na^99m^TcO_4_ accumulation) were 26, 6, and 0.8, respectively (Fig. 6C). sEVs had renal and fecal excretion of ~ 25% and ~ 2% ID/g, respectively, at 24 h (n = 3; Fig. 6D). SPECT/CT imaging (Additional file 1: Fig. S2A) showed a signal in the tumor area 24 h post-injection.

**Fig. 7:**
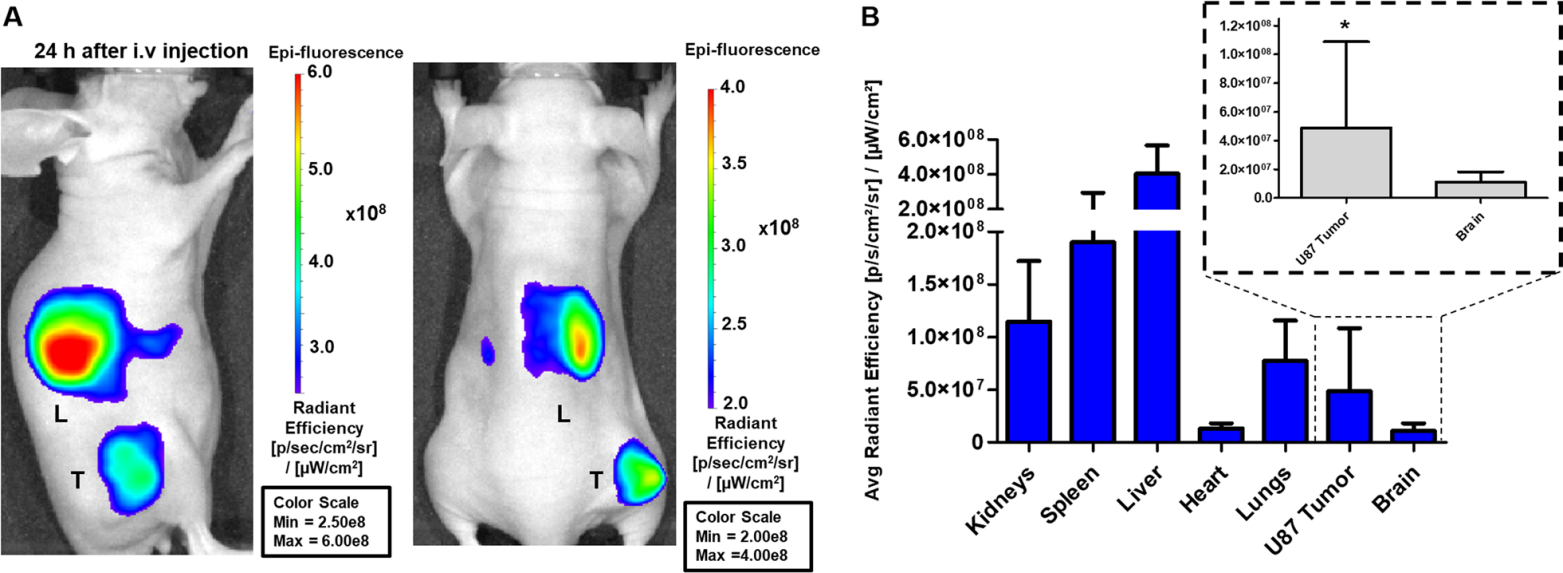
In vivo optical imaging of dual-sEVs. **A** In vivo optical imaging of tumor-bearing mice 24 h after i.v. injection in the lateral (left) and prone positions (right). **B** Ex vivo biodistribution of the excised organs (brain, spleen, kidneys, liver, tumor, heart, lungs; n = 11). Detailed quantification of the brain (control organ) compared to U87 tumor tissue. * *p* < 0.05. Data is represented as mean ± SD

In vivo fluorescence imaging showed a localized signal in the tumor area from 3 h post-injection (Additional file 1: Fig. S2B), which increased at 24 h (Fig. 7). Both 3 h and 24 h post-injection imaging showed a fluorescence signal in the liver area, whereas renal accumulation was found mainly at 3 h (Additional file 1: Fig. S2B). To confirm that the fluorescence was localized in the target organ, the animals were imaged again after removal of the tumor tissue and no fluorescence signals were found in the area (Additional file 1: Fig. S2C).

Quantification of the ex vivo biodistribution (Fig. 7B) resulted in a similar pattern as the nuclear quantification, with higher fluorescence in the liver (4.04 × 10^8^ ± 1.61 × 10^8^), spleen (1.90 × 10^8^ ± 1.05 × 10^8^), and kidneys (1.15 × 10^8^ ± 5.72 × 10^7^ [p/s/cm^2^/sr]/[ μW/cm^2^]). The glioblastoma tumor signal (4.89 × 10^7^ ± 5.99 × 10^7^) was compared to the control organ, the brain (1.44 × 10^7^ ± 7.14 × 10^6^ [p/s/cm^2^/sr]/[μW/cm^2^]), confirming significant differences (paired t-test; * *p* < 0.05).

### Histological evaluation of the tumor microenvironment and dual-SEV uptake

**Fig. 8:**
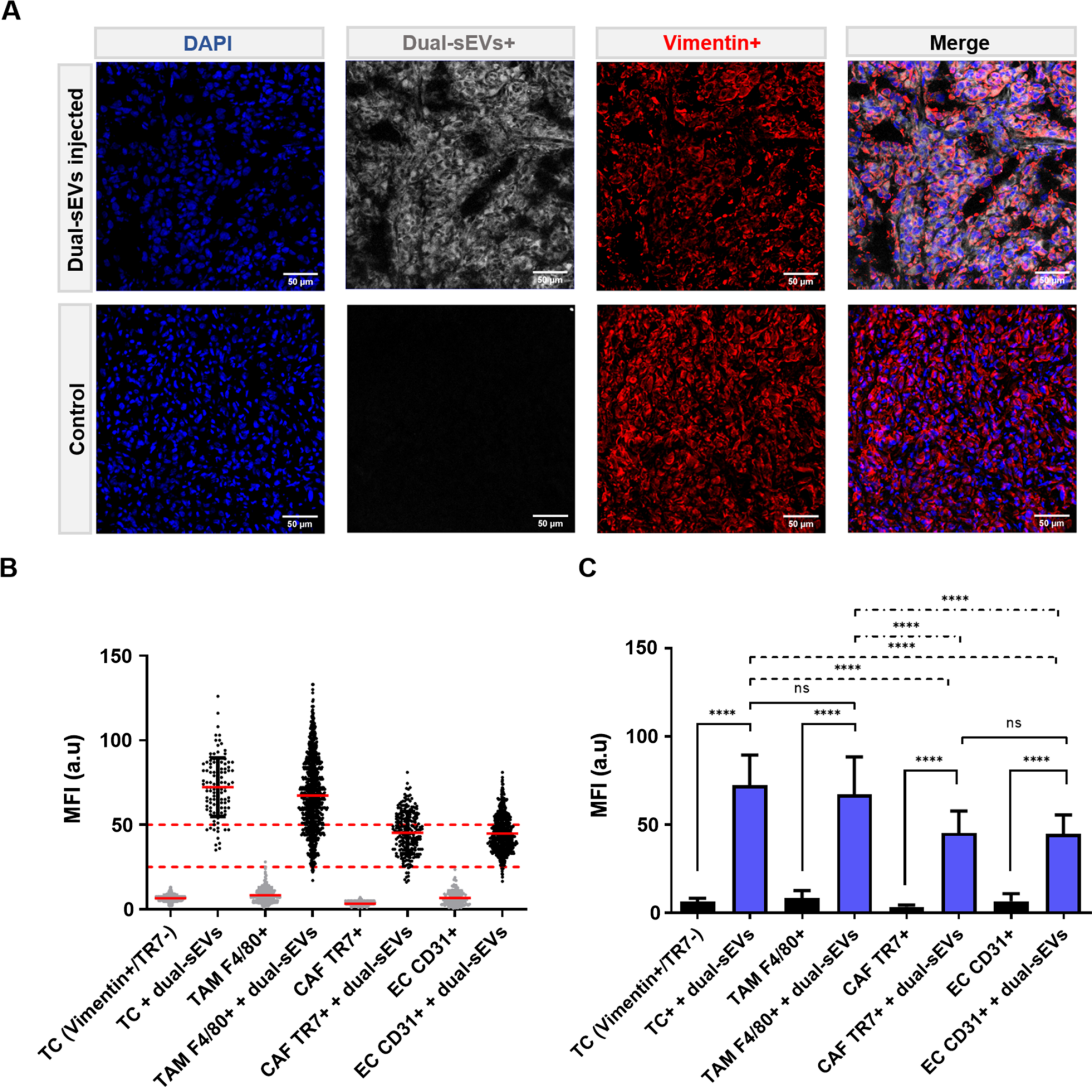
Histological analysis of the tumor microenvironment. **A** Confocal microscopy of tumor tissue with injection of dual-sEVs (white). Blue, DAPI; red, vimentin + . **B** Quantification of the uptake by control and injected (dual-sEVs +) populations: TCs, vimentin + /TR7-; TAMs, F4/80 + ; CAFs, TR7 + ; and ECs, CD31 + . **C** Mean uptake values in the populations. Data are represented as mean ± SD. **** *p* < 0.0001

Finally, histological analysis of tumor tissue (Fig. 8) allowed us to identify endothelial cells (ECs; CD31^+^), tumor-associated macrophages (TAMs; F4/80^+^), TCs (vimentin^+^ER-TR7^−^DAPI^+^), and cancer-associated fibroblasts (CAFs; ER-TR7^+^). Quantification of uptake confirmed TCs (U87 cells) and TAMs as the main cell types responsible for the uptake, with no significant differences between these two cells (Kruskal–Wallis test). However, the uptake of TCs and TAMs was significantly higher than that of CAFs or ECs (****p < 0.0001). CAFs and ECs had significantly higher MFI values compared to the control tissue.

## Discussion

In recent years, numerous studies have incorporated imaging agents into the structure of EVs to address their diagnostic capacity or provide a better understanding of the behavior of these nanoparticles after their administration as drug delivery systems [31]. Although these results have improved our understanding of the properties of these vesicles, there are still limitations in the spatial resolution, tissue penetration, pharmacokinetic behavior, or biological interactions at the cellular level that need to be addressed. In the current work, we developed a chemical approach for dual-labeling sEVs with the radioisotope ^99m^Tc and fluorescent dye SCy5 based on previous protocols developed by the group in the design of monomodal probes[22, 29]. In those works, the stability of each chemical methodology separately was demonstrated, showing radioactive and optical stabilities above 90% at 48 h. This novel dual approach was expected to provide a better understanding of the biological behavior of sEVs and their possible use in tumor detection.

Dual-sEVs combine the advantages of both techniques and provide us with deeper insight into the biological behavior of these vesicles intended as drug delivery systems. Though nuclear techniques have allowed an in-depth study of pharmacokinetic properties, optical techniques have confirmed their ability to localize tumor tissue in vivo and to determine the subpopulations in tumor tissues (TCs, TAMs, CAFs, and ECs) that preferentially uptake the probe.

From a chemical point of view, purity of our dual probe was confirmed by nuclear and UV–VIS HPLC (Fig. 2), in which only one peak was observed at a retention time of 25 min, in agreement with previous chromatogram data obtained with sEVs radiolabeling [22]. Furthermore, the HPLC chromatograms did not show any peaks corresponding to free SCy5 at ~ 75 min or unbound ^99m^TcO_2_ at ~ 90 min, proving the purity of the final dual nanosystem. In terms of vesicle morphology, TEM showed that the labeling methodology preserved the cup-shaped structure typical of sEVs, and TEM and NTA showed a slight size increase of 20 nm with respect to initial vesicles, likely explained by the fluorophore incorporation into the surface (Fig. 3). This phenomenon has already been described in other fluorescent sEVs labeled with SCy7.5 or BDP-FL [29].

These milk-derived vesicles were selected not only on the basis of their structural characteristics, such as nanometric size, robustness, and lipid bilayer morphology, but also on their proven role in tumor pathology [32–36] or inflammatory processes, as shown in previous studies using goat milk sEVs as optical probes in a model of peritonitis [37] or in a recent study where their anti-viral activity was probed [38]. Moreover, milk EVs from other sources have been proposed as an excellent platform for drug delivery in cancer therapy [35], further supporting our application of these vesicles in the detection of tumor foci.

To assess our dual-sEVs in tumor processes and inflammatory response, we conducted an in vitro study using myeloid RAW 264.7 cells, together with two well-known human cancer cell lines, U87 and HeLa. Both fluorescence and radioactivity studies showed RAW 264.7 cells to have the highest uptake of dual-sEVs. Similar interactions and high uptake between sEVs and these cells were described previously for other vesicles of lactic origin [37]. The lower uptake of the probe in this tumor cell line compared to RAW 264.7 could be due to a lower metabolic activity and higher doubling time (34 h vs 21 h). HeLa cells presented uptake saturation after 1 h, with no further increase with time, thus supporting their use as control cells. To the authors knowledge, there is no previous literature that support the existence of interaction between milk EV and HeLa cells. Based on these results, the ability of the dual-sEVs to detect in vivo these cells population was tested in a xenograft tumor model.

The pharmacokinetic study was carried out by taking advantage of the high sensitivity of nuclear techniques. Radioactive quantification by a gamma counter confirmed a long t_1/2_ β of almost 8 h in circulation. These pharmacokinetics seem to outperform conventional liposome-based systems, which present a lower t_1/2_ β [39] and could facilitate higher tumor accumulation as described elsewhere [40]. Moreover, the qualitative and quantitative assessment by in vivo SPECT/CT imaging and ex vivo gamma counter showed main accumulation in liver and spleen, also observed in previous in vivo assessment of EVs after intravenous administration [22, 29, 41, 42], which corresponds with nanoparticles of this size and morphology [43]. The tumor/control organ (brain) ratio (Fig. 6C) showed a high tumor to healthy tissue ratio, supporting further evaluation of the probe in a more realistic orthotopic glioblastoma model. Interestingly, the trachea uptake, which is commonly studied in pharmacokinetics studies as the main organ of free ^99m^Tc accumulation, did not reach 0.5% ID/g, which indicates high stability of the probe (Fig. 6B).

Finally, the diagnostic capability of our imaging agent was validated by optical imaging. In vivo fluorescence imaging of dual-sEVs showed a clear signal in the tumor area 3 h after intravenous (i.v.) injection (Additional file 1: Fig. S2A). The uptake observed short-term suggests incorporation into the tumor tissue due to specific affinity related to tumor or inflammatory processes because these time values are still too short to be attributed to the enhanced permeability and retention (EPR) phenomenon, which is mainly observed 24 h after administration of the nanomaterials [44]. Therefore, the increase in tumor uptake observed longer term (24 h) could be partially explained by an additional EPR effect and/or the presence of more inflammatory cells (e.g., macrophages) within the tissue (Fig. 7). This EPR effect has been well described for nanoparticles with a hydrodynamic size < 200 nm [44, 45].

Ex vivo optical imaging of the harvested organs (Additional file 1: Fig. S2D) confirmed nanoprobe accumulation in secretory and reticuloendothelial organs (liver, spleen, and kidneys), similar to the biological behavior observed by in vivo optical imaging. Tissue-resident macrophages are present at high numbers in these organs and are responsible for the clearance of EVs [40].

Finally, a histological study (Fig. 8) allowed us to characterize the tumor environment and the specific cell populations involved in the uptake of sEVs. We used different markers to identify the main populations (U87 TCs, TAMs, CAFs, and ECs) that could be responsible for the uptake. U87 cells were marked using vimentin, as it is expressed in glioblastoma cells when the tumor presents stemness characteristics and can be differentiated from CAFs due to being negative for ER-TR7 (Additional file 1: Fig. S3). Vimentin has been detected in neurospheres and not in cultured cells, and it is associated with more aggressive tumorigenicity [46]. This evaluation at a cellular level showed that the main populations responsible for the uptake of dual-sEVs were the human glioblastoma cancer cells and TAMs. The involvement of TAMs in tumor development is well-known [47] and is a key target for cancer therapy [48]. On the other hand, ECs and CAFs are also responsible for the sEVs incorporation into the tumor, at a much smaller scale, probably due to the fact that the vasculature is a point of entry for the nanoprobe after i.v. administration. The optical properties of the vesicles allowed us to assess their interaction at the cellular and tissue level with the different populations in the tumor microenvironment. All of this information will eventually translate into a better understanding of the natural compartmentalization and intercommunication of the dual-sEVs which may ultimately facilitate their promising clinical transfer as a theragnostic platform.

Our study has some limitations. First, the use of PET isotopes appears to be a better alternative to using ^99m^Tc due to the weak signal obtained from the tumor tissue, poor spatial resolution of SPECT, and difficulty providing reliable quantification. Among the possible PET isotopes, the following may be promising alternatives: radiometals with similar coordination chemistry, such as ^64^Cu (t_1/2_ = 12 h) and ^89^Zr (t_1/2_ = 78 h), or radioisotopes such as ^124^I (t_1/2_ = 4 days) [49, 50]. Different strategies can improve the image resolution based on the increase in specific activity with radiolabeling. One of the main approaches in radiolabeling nanoparticles is the incorporation of a chelating agent (e.g., DTPA, DFO) on the surface of the nanoparticle [51], which promotes the coordination and incorporation of the radiometal (e.g., ^64^Cu or ^89^Zr) into the nanostructure. Another strategy to obtain a higher specific activity could be the selective reaction of ^124^I with the protein's tyrosine groups present on the sEVs surface. Second, as demonstrated by both optical and nuclear techniques in our study, the biological behavior of our vesicles leads to high uptake by the reticuloendothelial system (liver, spleen). This uptake may lead to secondary toxicity in these organs or to false negatives in the case of processes associated with liver pathology. New methodologies based on bioenrichment of the EV surface could be implemented to selectively increase accumulation in the target tissue (tumor) compared to liver tissue, overcoming the previous limitation. Third, the sample sizes in the in vivo studies could be increased to obtain significant differences or evaluate the nanoprobe at different time points. Finally, the use of subcutaneous models does not allow for the assessment of problems derived from the blood–brain barrier. Therefore, future studies should include evaluation of our probe in orthotopic models. These models would confirm its biodistribution and the ability to discern between healthy brain tissue and tumor tissue.

## Conclusions

We have developed a dual imaging agent based on radioactive and optical labeling of natural sEVs. This novel dual approach provides further insight into the biological behavior of these EVs. Though nuclear techniques enable quantitative analysis of the pharmacokinetic properties of sEVs, optical studies confirmed their ability to target tumor tissue and allowed detailed identification of the cell subpopulations involved in their uptake. We also evaluated the biological behavior of the new nanoprobe in vivo and in vitro*,* showing the ability of dual-sEVs to identify malignant glioblastoma tumor tissue in vivo.

## Methods

The study aims to validate the application of dual-sEVs as imaging nanosystems in oncology. For this purpose, a fully physicochemical characterization was conducted using nanometric and chromatographic techniques. Afterwards, in vitro uptake studies were carried out in different tumor cell lines detecting nuclear and optical signals. Finally, in vivo non-invasive PET/CT and optical imaging were employed to validate the nanoprobe in a mouse model.

### sEVs extraction

sEVs were extracted and purified from goat semi-skimmed milk (El Cantero de Letur, Albacete, Spain) by ultracentrifugation based on previous protocols [22, 29]. We utilized differential centrifugation and PD-10 size exclusion columns (GE Healthcare Bio-Sciences AB, Chicago, IL, USA). The SEV protein content was quantified by Bradford Coomassie protein assay.

### Synthesis of dual-sEVs

Commercial sodium pertechnetate (Na^99m^TcO_4_) was obtained from ^99^Mo/^99m^Tc TECKIS™ Technetium ^99m^Tc Generator (Curium Pharma, Madrid, Spain). Freshly eluted Na^99m^TcO_4_ (15 µL, 2 mCi) was reduced with 15 µL of 2 mM stannous chloride anhydride (SnCl_2_ 2H_2_O) in acetic acid (AcOH; 10%V; Sigma-Aldrich, St. Louis, MO) to form technetium oxide (^99m^TcO_2_). The reaction was carried out for 5 min at 37 °C under an N_2_ atmosphere. Next, 90 µg of goat’s milk sEVs in 1X phosphate buffered saline (PBS; Gibco, ThermoFisher Scientific, Waltham, MA, USA) were mixed with ^99m^TcO_2_ for 30 min at 37 °C at physiological pH [22]. The ^99m^Tc-sEVs were then mixed with 10 μL of 16.9 mM SCy5 (Lumiprobe, GmbH, Hannover Germany) for 60 min at 37 °C at physiological pH. The final product, dual-sEVs, was purified using Exosome Spin Columns (MW 3000; Invitrogen™, Carlsbad, CA, USA) in each step of the reaction.

Specific activity of dual-sEVs was calculated in based of the final activity of the sample considering the initial concentration of the sEVs (90 µg) in the reaction,

### Fluorometry

Fluorophore concentration and fluorescence emission spectrum of dual-sEVs were obtained with a NanoDrop™ Fluorometer 3300 (ThermoFisher Scientific, Waltham, MA, USA) using a SCy5 standard curve (0.5–16.9 μM) and 665 nm emission filter.

### Nanoparticle tracking analysis

A NanoSight NS300 (Malvern Instruments, Ltd, UK) equipped with a high sensitivity sCMOS camera was used to measure the real-time concentration (particles/mL) and size distribution of sEVs in suspension. The sample was evaluated at 25 °C: viscosity 0.9 cP (water), 20–30 for threshold, and 7 for screen gain. Five 60-s videos were recorded per sample (screen gain 1; camera level 11 or 13; 60–80 frames/s particle movement) and analyzed by NTA v3.4 software.

### Transmission electron microscopy

The morphological characteristics of the sEVs after labeling were analyzed by transmission electron microscopy (JEOL JEM-1010 from *ICTS* Centro *Nacional de Microscopía Electrónica;* Universidad Complutense de Madrid, Spain). Dual-sEVs nanoparticles were negatively stained with 2% uranyl acetate at room temperature and their images acquired using a Megaview II digital camera and processed by DigitalMicrograph™ software.

### High performance liquid chromatography

The purity of labeled sEVs was confirmed by HPLC on an Agilent 1200 series (Agilent Technologies, Santa Clara, CA, USA) equipped with a UV–VIS detector (254 and 600 nm) and a MIRA * μ-HPLC radioactivity flow detector (Elysia-Raytest, Angleur, Belgium) and SEC-3000 column (300 × 7.8 mm; Phenomenex, Inc., Torrance, CA, USA). An isocratic gradient of 1X PBS with a flow rate of 0.2 mL/min for 90 min was employed. Gina Star (Microbeam S.A., Madrid, Spain) Chromatography Software was employed for data acquisition, evaluation, integration, and system control.

### Radio thin layer chromatography

The radiochemical purity (RCP) of the probe was evaluated by radio-TLC analysis (stationary phase: silica gel 60 F_254_ aluminum sheets; mobile phases: acetone and 0.9% NaCl). To evaluate the RCP of the dual-sEVs, 3 µL of labeled sEVs were deposited 1 cm apart on 15 × 100 mm TLC plates (silica gel 60 F_254_ aluminum sheets, Merck, Germany) and developed with 100% acetone. After developing, the plates were dried at room temperature and scanned with a MiniGina Single TLC system (Elysia-Raytest, Angleur, Belgium) for 5 min. The resulting chromatograms were analyzed by GINA-STAR software (Elysia-Raytest).

### Cell culture

All cell lines were grown in Dulbecco's Modified Eagle Medium (DMEM; D6429, Sigma Aldrich, St. Louis, MO) supplemented with 10% fetal bovine serum (FBS, Gibco®, 10,270, centrifuged 18 h/100,000 × *g*/4 °C) and 1% penicillin, streptomycin, and amphotericin B (Lonza, 17-745H). Cells were cultured at 37 °C in a 5% CO_2_ atmosphere.

The murine RAW 264.7 (ATCC® TIB-71™) cell line was used as a model of the inflammatory response mediated by macrophages. For maintenance of the cell line, the medium was changed every other day and cells subcultured in 25 cm^2^ cell culture flasks (Corning® Costar, NY, USA) with a cell scraper (Corning® Costar, NY, USA).

The human HeLa (ATCC® CCL-2™) cell line was used as a model of cervical carcinoma and human U87 (ATCC® HTB-14™) cell line as a model of glioblastoma. For maintenance of the cell lines, the medium was changed 2 to 3 times per week and cells subcultured in 25 cm^2^ flasks with trypsin–EDTA solution (Sigma Aldrich, St. Louis, MO).

### Radioactive in vitro uptake of dual-sEVs

Quantitative radioactive assessment of the radiotracer uptake was performed in RAW 264.7, HeLa, and U87 cells. In this study, cells were plated on 12-well plates (Corning® Costar, NY, USA) with SEV-free complete DMEM. A total of 20 µCi of dual-sEVs (1.25 µg/mL, 1.85 × 10^8^ ± 1.99 × 10^7^ particles/mL) were added to the cells and incubated for 1 h, 4 h, and 24 h. After these time points, the DMEM was removed, and cells carefully washed twice with 1 mL of 1X PBS. Cells were trypsinized with trypsin/EDTA for 10 min and collected. Finally, the radioactive activity of DMEM, 1X PBS supernatant, and cells were measured on a 2470 Wizard2™ Gamma Counter (Perkin Elmer, USA). Parallel control assessments were performed employing commercial Na^99m^TcO_4_ (~ 20 µCi).

### In vitro uptake of dual-sEVs by confocal imaging

Qualitative fluorescent assessment of dual-sEVs was performed in RAW 264.7, HeLa, and U87 cells by confocal microscopy. Cells were plated on 24-well plates (Corning® Costar, NY, USA) over glass coverslips at a cell density of 1.5 × 10^4^ cells/cm^2^ for RAW 264.7 cells or 1 × 10^4^ cells/cm^2^ for HeLa and U87 cells in SEV-depleted complete DMEM. Next, 5 µg/mL (3.70 × 10^8^ ± 3.98 × 10^7^ particles/mL) dual-sEVs were added at 5 min, 1 h, 4 h, and 24 h. After these time points, cells were fixed with 2% formaldehyde solution for 10 min, and their filament actin cytoskeleton was stained with Phalloidin-iFluor 555 Reagent (Abcam, ab176756) and their nuclei with 4′,6-diamidino-2-phenylindole (DAPI; Sigma-Aldrich, St. Louis, MO). Dako Fluorescence Mounting Medium (Sigma-Aldrich, St. Louis, MO) was used to prepare the coverslips. Cells were observed under a confocal microscope (Leica TCS SPE; Leica Microsystems Inc., Buffalo Grove, IL, USA) from *Unidad de Medicina y Cirugía Experimental* (Instituto de Investigación Sanitaria Gregorio Marañón, Madrid, Spain).

### In vitro uptake of dual-sEVs by flow cytometry

Quantitative assessment of dual-sEVs was performed in RAW 264.7, HeLa, and U87 cells by flow cytometry. Cells were plated on 12-well plates at a cell density of 1.5 × 10^4^ cells/cm^2^ for RAW 264.7 cells or 1 × 10^4^ cells/cm^2^ for HeLa and U87 cells in sEVs-depleted complete DMEM. Next, 5 µg/mL (3.70 × 10^8^ ± 3.98 × 10^7^ particles/mL) dual-sEVs were added at 5 min, 1 h, 4 h, and 24 h. After these time points, cells were trypsinized with trypsin/EDTA for 5 min and the cells collected. Analysis was performed using a Gallios Flow Cytometer (Beckman Coulter Instruments, Brea, CA, USA) from *Unidad de Medicina y Cirugía Experimental* (Instituto de Investigación Sanitaria Gregorio Marañón, Madrid, Spain). A red laser (λ_ex_ = 633 nm detection FL6, λ_em_ = 660/20 nm) was used to identify dual-SEV uptake. The same laser conditions were applied for all cell types. Data were analyzed in FlowJo™ (Ashland) v10.7 software. Doublets were discriminated by forward scatter height and area (FSC-H/FSC-A), and cells were identified by side scatter and forward scatter area (SSC-A/FSC-A). MFI was calculated with the geometric mean.

### Glioblastoma mouse model

We used 6 to 8-week-old female athymic nude mice [CR ATH HO; Crl:NU(NCr)-*Foxn*1^nu^, Charles River Laboratories, France] for all mouse experiments. The harvested U87 cells were suspended in a 1:1 (v/v) mixture of complete DMEM/Matrigel (Corning™ 354,234, NY, USA) and injected subcutaneously (2 × 10^6^ cells in 150 µL) into the right rear flanks of the mice. The mice were anesthetized by inhalation of a 2% sevoflurane/oxygen gas mixture before the procedure. Animals were checked daily post-inoculation and tumor measurements were made every 3 days. Mice were used for studies when the tumors reached 40–90 mm^3^ in size.

All experimental procedures with animals conformed to EU Directive 2010/63EU and Recommendation 2007/526/EC found in RD 53/2013. Animal protocols were approved by the *Comité de Ética en Experimentación Animal del Hospital Gregorio Marañón* and the Animal Protection Area of the Comunidad Autónoma de Madrid (PROEX 097–016). The 4 to 5-week-old female athymic nude CR ATH HO mice were left for 2 weeks to acclimatize and maintained in sealed cages with HEPA-filtered air on a 12-h light cycle and food and water ad libitum.

### Blood half-life and blood clearance of dual-sEVs

The T_1/2_ β and CL of dual-sEVs were determined in female nude mice (same strain, n = 5) by measuring activity in serial blood samplings after i.v. injection of dual-SEV nanoparticles into the tail vein (200–300 µCi, 30 µg in 200 µL 1X PBS). Blood samples were extracted from the tails of awake mice at several time points post-injection (5, 10, 15, 20, 25, 40, 55, 85, 120, 140, 1075, 1240, 1305 min). The radioactivity of the samples was measured on a 2470 Wizard2™ Gamma Counter (Perkin Elmer, USA) and presented as the mean of percent injected per milliliter of sample (% ID/mL). The values were calculated using PKSolver software and an add-in program for Microsoft Excel with a two-compartmental kinetic analysis model.

### In vivo SPECT/CT imaging of dual-sEVs

In vivo SPECT /CT imaging was performed in athymic nude mice (n = 3) employing the MiLabs USPECT II (the Netherlands, EU) and CT PET/CT SuperArgus (SEDECAL, Spain) systems. To register the SPECT and CT images, each animal was placed on a homemade multimodal bed surrounded by three capillaries filled with a mixture of ^99m^Tc and iopamiro, which were visible in both modalities. The spatial transformation to align SPECT and CT images was achieved by matching the corresponding fiducials of both modalities using a method analogous to that described by Cussó et al. [52]. Dual-sEV nanoparticles were injected i.v. into the tail vein (200–300 µCi, 30 µg, 200 µL 1X PBS) of nude female mice (n = 3). Animals were imaged under 2% isoflurane anesthesia in 100% O_2_ and the field of view adjusted to the area of interest. SPECT images were acquired 24 h after the radiotracer was administered (1 static frame of 60 min, fast dynamic with 24 volumes) using a multi pinhole collimator and an energy window ranging from 126 to 154 keV. OS-EM reconstruction was performed with a 0.75-mm^3^ voxel size, 16 subsets, and 2 iterations using proprietary software (MiLabs, the Netherlands, EU). Anatomical CT images were acquired with an X-ray beam current of 340 µA and tube voltage of 40 kVp, 360 projections, and 2 × 2 binning. Images were reconstructed using the Feldkamp, Davis, and Kres (FDK) algorithm [53]. Animals used for SPECT/CT imaging were also evaluated by optical IVIS imaging.

### Ex vivo biodistribution

Twenty-four hours after i.v. injection of the probe and after in vivo imaging, mice (n = 3) were sacrificed and their major organs (tumor, trachea/thyroid, lungs, heart, liver, spleen, kidneys, intestines, muscle, and brain), as well as blood, urine, and feces, were collected, weighed, and placed in scintillation vials. The activity in the organs of interest was measured on a 2470 Wizard2™ Gamma Counter (Perkin Elmer, USA). Radioactivity readings (counts per minute—CPM) were expressed as the percentage of injected dose per gram of tissue (% ID/g).

### Optical IVIS imaging of dual-sEVs

In vivo probe uptake was evaluated by optical imaging using an IVIS® Lumina III in vivo imaging system (Perkin Elmer, Waltham, MA) employing a spectral unmixing protocol for the Cy5 fluorophore. Mice were anesthetized with 2% isoflurane in 100% O_2_ via a facemask during the whole in vivo imaging procedure with an XGI-8 anesthesia system (100 V). Animals were placed in the supine, prone, and lateral positions and images taken 3 h and 24 h after i.v. injection of the dual probe (30 µg/200 µL, 2.22 × 10^9^ ± 2.38 × 10^8^ particles, 1X PBS, n = 11).

Images were analyzed and quantified using Living Image® 4.4 software (Perkin Elmer, Waltham, MA). Ex vivo quantification (n = 11) was performed after the organs of interest (tumor, brain, liver, spleen, heart, kidneys, and lungs) were excised and quantified by the average radiant efficiency. Data were expressed as mean ± SD in (p/s/cm^2^/sr)/(μW/cm^2^).

### Immunohistochemistry

After the last in vivo imaging time point (24 h), U87 tumors and organs (liver, kidneys, and spleen) were harvested (n = 3) and embedded in Tissue-Tek® O.C.T. (Sakura), frozen quickly with 2-methylbutane (Sigma-Aldrich, St. Louis, MO), and maintained at −80 °C. Sections were cut using a cryostat (Leica CM1950; Leica Microsystems Inc., Buffalo Grove, IL, USA) at 8 µm, fixed with cold acetone for 5 min, and stored at −20 °C. The tumor microenvironment in glioblastoma xenografts was analyzed by immunofluorescence (IF), staining with DAPI (0.2 μg/mL) and the following antibodies: rabbit anti-vimentin antibody (2.68 μg/mL, ab92547, Abcam), secondary antibody Alexa Fluor 488 chicken anti-rabbit (4 μg/mL, A-21441, Life Invitrogen); Alexa Fluor 488 Rat anti-mouse F4/80 antibody (10 μg/mL, 123,120, Biolegend); Armenian hamster anti-CD31 antibody (6.7µg/mL, MA3105, Invitrogen) and Alexa Fluor 594 goat anti-hamster (2.5 μg/mL, 405,512, Biolegend); rat anti-reticular fibroblast and reticular fiber antibody (4 μg/mL, ab51824, Abcam) and cyanine3 goat anti-rat (2.5 μg/mL, 405,408, Biolegend). Primary antibodies were incubated for 1 h at room temperature in the dark, followed by 1 h incubation with the respective secondary antibodies under the same conditions. Cryo-tissues were coverslipped with Dako and observed using a confocal microscope (Leica TCS SPE).

### Uptake quantification by confocal microscopy

U87 tumors stained with different antibodies were analyzed by confocal microscopy. TCs were marked with anti-vimentin antibody, TAMs with anti-F4/80 antibody, ECs with CD31 antibody, and CAFs with ER-TR7 antibody. Several 20 × fields (n = 3) were analyzed at regions of interest to measure the MFI of the dual-sEVs. For in vivo quantification of dual-sEVs in TCs, vimentin, ER-TR7, and DAPI-stained cells (vimentin^+^ ER^−^TR7^−^ DAPI^+^) were segmented and the MFI of dual-sEVs quantified at matched single cells [54]. Similarly, the MFI was quantified in TAMs (F4/80^+^ DAPI^+^), ECs (CD31^+^ DAPI^+^), and CAFs (ER-TR7^+^ DAPI^+^). Dual-sEVs uptake by cells in the tumor microenvironment was quantified using FIJI software.

### Statistical analysis

Data were represented as mean ± SD and analyzed using Prism software 6.01 (Graph pad, Inc.). For in vitro fluorescent uptake, one-way ANOVA and Tukey’s post-test were performed. For in vivo optical and nuclear biodistribution, a paired t-test was used. For histological quantification of the probe uptake, results were non-Gaussian and the Kruskal–Wallis test and Dunn’s multiple comparisons tests were performed. For all statistical analyses, *P*-values < 0.05 were considered significant.

## Supplementary Information

**Additional file 1**. **Fig. S1**. Physicochemical characterization of non-labeled sEVs by transmission electron microscopy. Images showing the morphology and size of unlabeled sEVs. **Fig. S2**. In vivo and ex vivo studies of dual-sEVs. **A** In vivo SPECT/CT imaging of a tumor 24 h after i.v injection of the dual-sEVs. **B** In vivo optical imaging of tumor-bearing mice 3 h after i.v. injection in the lateral (left) and prone (right) positions. **C** Ex vivo optical imaging of mice without skin with the tumor exposed (left) and with excised skin and tumor (right). **D** Ex vivo optical imaging of excised organs (brain, spleen, kidneys, tumor, heart, lungs) from control mice without nanoprobe injection and mice injected with dual-sEVs. **Fig. S3**. Confocal microscopy of the histological analysis of the tumor microenvironment population with injection of dual-sEVs (white). Blue, DAPI; red, F4/80+ (TAMs), CD31+ (ECs), and ER-TR7+ (CAFs). **Fig. S4**. Confocal microscopy of the histological analysis of the U87 tumor cell phenotype. Blue, DAPI; white, dual-sEVs; red, ER-TR7- (CAFs), and green, vimentin+ (U87).

## Data Availability

The datasets used and/or analyzed during the current study are available from the corresponding author on reasonable request.

## Abbreviations

CL: Blood clearance
t_1/2_: Blood half-life
CAF: Cancer-associated fibroblast
CPM: Counts per minute
CPS: Counts per second
DMEM: Dulbecco’s Modified Eagle Medium
DAPI: 4′,6-diamidino-2-phenylindole
DDS: Drug delivery systems
EC: Endothelial cell
EPR: Enhanced permeability and retention
EV: Extracellular vesicle
FBS: Fetal bovine serum
HPLC: High-performance liquid chromatography
IF: Immunofluorescence
MFI: Median fluorescence intensity
NHS: *N-*Hydroxysuccinimide
NTA: Nanoparticle tracking analysis
PBS: Phosphate buffered saline
PET: Positron emission tomography
RFU: Relative fluorescent units
SPECT: Single photon emission computed tomography
sEVs: Small extracellular vesicles
SD: Standard deviation
SCy5: Sulfo-cyanine5 NHS ester
^99m^Tc: Technetium-99 metastable
TLC: Thin layer chromatography
TEM: Transmission electron microscopy
TAM: Tumor-associated macrophage
TCs: Tumor cells
