# Supplementary material for: Dual-labeled nanoparticles based on small extracellular vesicles for tumor detection
Source: Biol Direct. 2022 Nov 14;17:31. doi: 10.1186/s13062-022-00345-7 (PMC9664624; doi:10.1186/s13062-022-00345-7)
Supplement: Supplementary file 1 — Additional file 1. Fig. S1. Physicochemical characterization of non-labeled sEVs by transmission electron microscopy. Images showing the morphology and size of unlabeled sEVs. Fig. S2. In vivo and ex vivo studies of dual-sEVs. A In vivo SPECT/CT imaging of a tumor 24 h after i.v injection of the dual-sEVs. B In vivo optical imaging of tumor-bearing mice 3 h after i.v. injection in the lateral (left) and prone (right) positions. C Ex vivo optical imaging of mice without skin with the tumor exposed (left) and with excised skin and tumor (right). D Ex vivo optical imaging of excised organs (brain, spleen, kidneys, tumor, heart, lungs) from control mice without nanoprobe injection and mice injected with dual-sEVs. Fig. S3. Confocal microscopy of the histological analysis of the tumor microenvironment population with injection of dual-sEVs (white). Blue, DAPI; red, F4/80+ (TAMs), CD31+ (ECs), and ER-TR7+ (CAFs). Fig. S4. Confocal microscopy of the histological analysis of the U87 tumor cell phenotype. Blue, DAPI; white, dual-sEVs; red, ER-TR7- (CAFs), and green, vimentin+ (U87). [file 13062_2022_345_MOESM1_ESM.docx]

**Supplementary material**

**
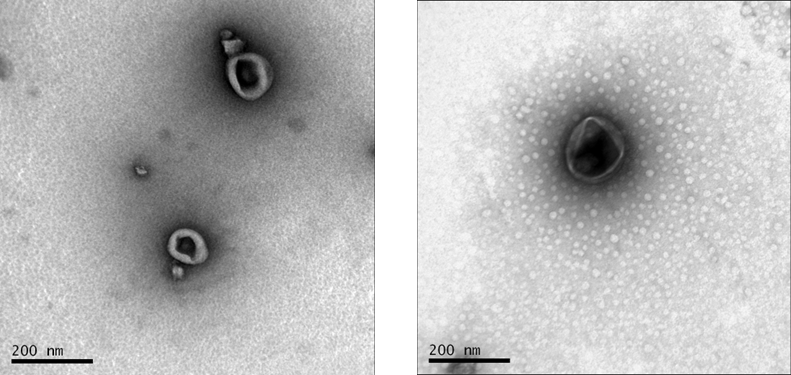
**

**Supplementary Figure 1.** Physicochemical characterization of non-labeled sEVs by transmission electron microscopy. Images showing the morphology and size of unlabeled sEVs.


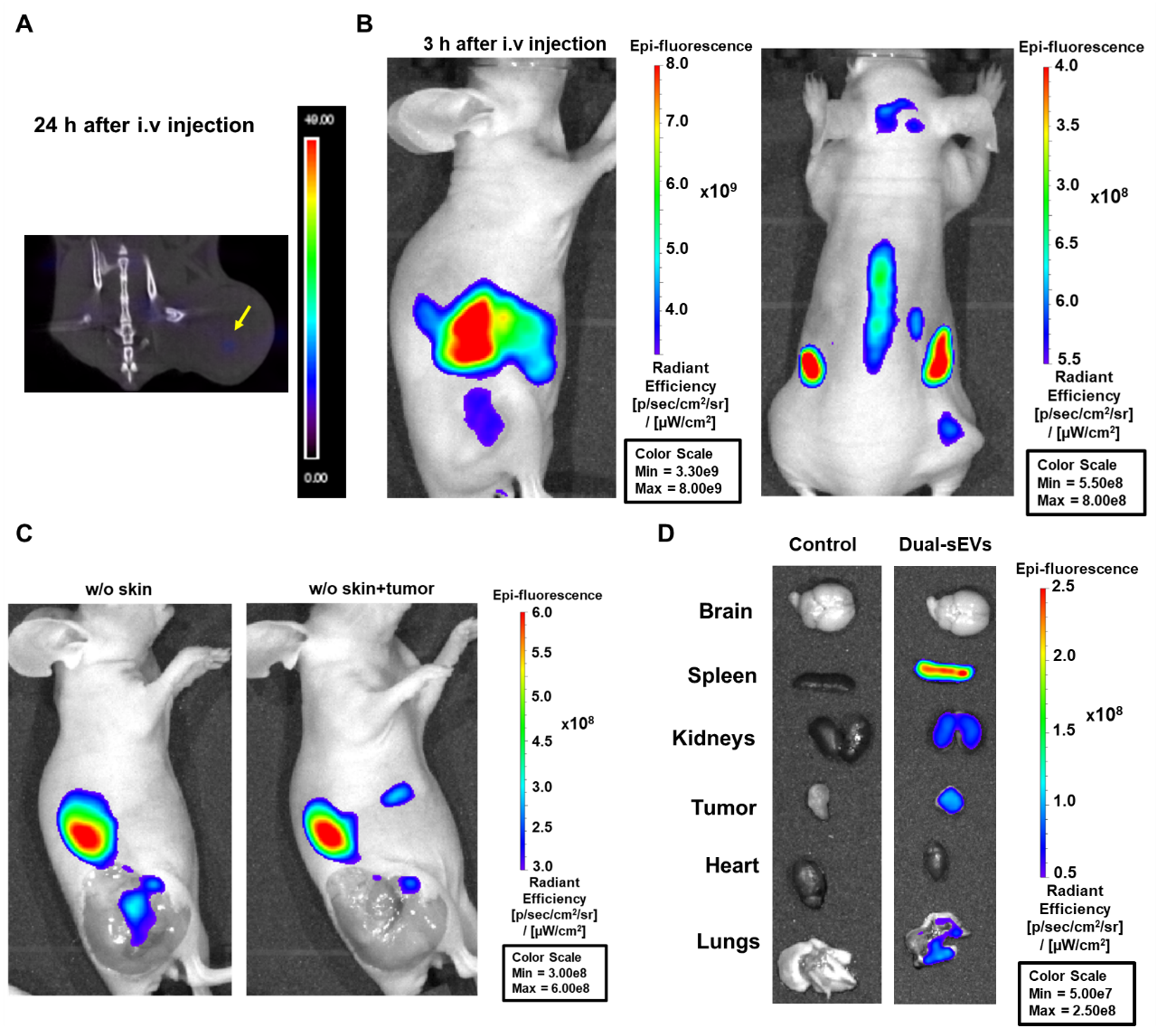


**Supplementary Figure 2**. *In vivo* and *ex vivo* studies of dual-sEVs. A. *In vivo* SPECT/CT imaging of a tumor 24 h after i.v injection of the dual-sEVs. B. *In vivo* optical imaging of tumor-bearing mice 3 h after i.v. injection in the lateral (left) and prone (right) positions. C. *Ex vivo* optical imaging of mice without skin with the tumor exposed (left) and with excised skin and tumor (right). D. *Ex vivo* optical imaging of excised organs (brain, spleen, kidneys, tumor, heart, lungs) from control mice without nanoprobe injection and mice injected with dual-sEVs.

**
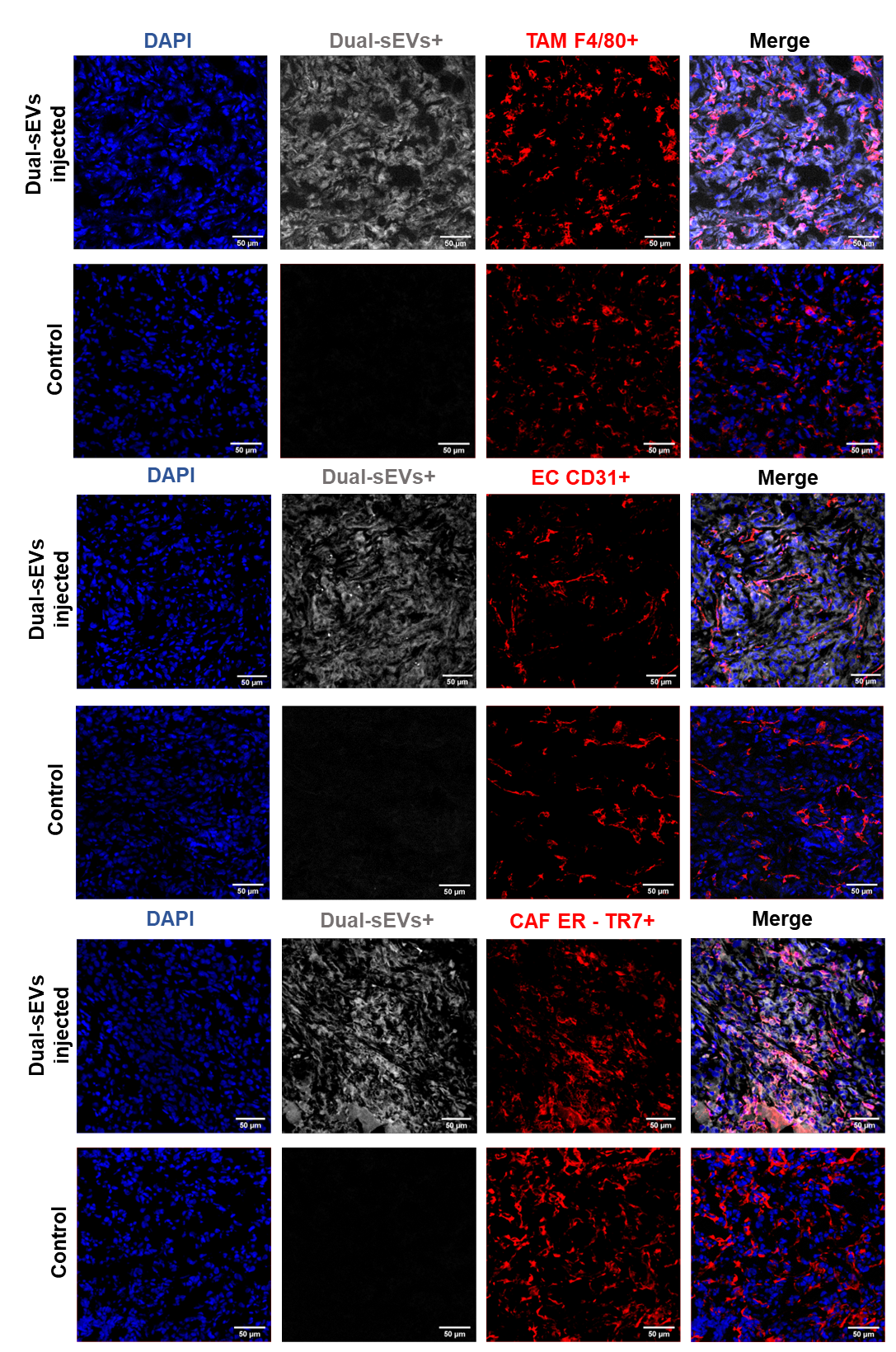
**

**Supplementary Figure 3.** Confocal microscopy of the histological analysis of the tumor microenvironment population with injection of dual-sEVs (white). Blue, DAPI; red, F4/80+ (TAMs), CD31+ (ECs), and ER-TR7+ (CAFs).

**
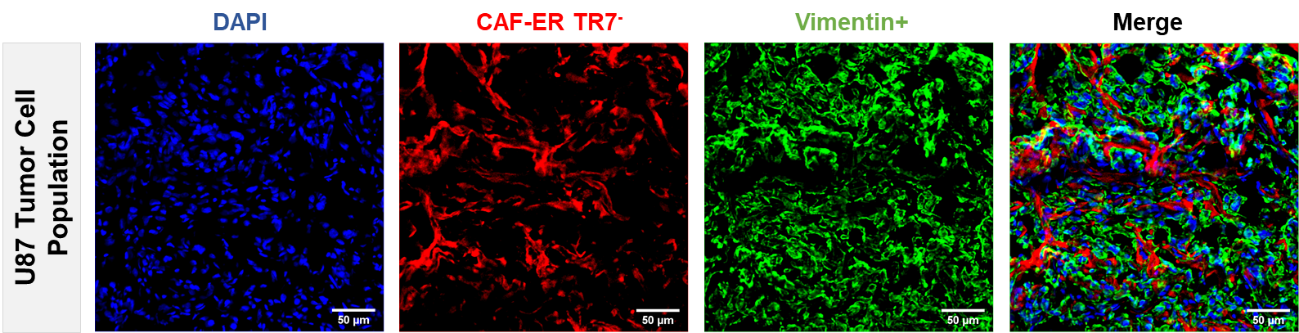
**

**Supplementary Figure 4.** Confocal microscopy of the histological analysis of the U87 tumor cell phenotype. Blue, DAPI; white, dual-sEVs; red, ER-TR7^-^ (CAFs), and green, vimentin+ (U87).
